# Lifestyle risk score and mortality in Korean adults: a population-based cohort study

**DOI:** 10.1038/s41598-020-66742-y

**Published:** 2020-06-24

**Authors:** Dong Hoon Lee, Jin Young Nam, Sohyeon Kwon, NaNa Keum, Jong-Tae Lee, Min-Jeong Shin, Hannah Oh

**Affiliations:** 1 Department of Nutrition, Harvard T.H. Chan School of Public Health, Boston, MA USA; 2 Department of Public Health Sciences, BK21PLUSProgram in Embodiment: Health-Society Interaction, Graduate School, Korea University, Seoul, Republic of Korea; 3 Department of Food Science and Biotechnology, Dongguk University, Goyang, South Korea; 4 Division of Health Policy and Management, College of Health Sciences, Korea University, Seoul, Republic of Korea; 5 Division of Biosystems and Biomedical Sciences, College of Health Sciences, Korea University, Seoul, Republic of Korea

**Keywords:** Diseases, Medical research, Risk factors

## Abstract

Individual lifestyle risk factors have been associated with an increased risk of mortality. However, limited evidence is available on the combined association of lifestyle risk factors with mortality in non-Western populations. The analysis included 37,472 participants (aged ≥19 years) in the Korea National Health and Nutrition Examination Surveys (2007–2014) for whom the data were linked to death certificates/medical records through December 2016. A lifestyle risk score was created using five unhealthy behaviors: current smoking, high-risk alcohol drinking, unhealthy weight, physical inactivity, and insufficient/prolonged sleep. Cox proportional hazards models were used to estimate hazard ratio (HR) and 95% confidence interval (CI). During up to 9 years of follow-up, we documented 1,057 total deaths. Compared to individuals with zero lifestyle risk factor, those with 4–5 lifestyle risk factors had 2.01 times (HR = 2.01, 95% CI = 1.43–2.82) and 2.59 times (HR = 2.59, 95% CI = 1.24–5.40) higher risk of all-cause and cardiovascular mortality, respectively. However, higher lifestyle risk score was not significantly associated with cancer mortality (p-trend >0.05). In stratified analyses, the positive associations tended to be stronger in adults aged <65 years, unemployed, and those with lower levels of education. In conclusion, combined unhealthy lifestyle behaviors were associated with substantially increased risk of total and cardiovascular mortality in Korean adults.

## Introduction

Individual lifestyle risk factors such as obesity, physical inactivity, smoking, heavy alcohol use and poor diet have been associated with increased risk of various chronic diseases and premature death^1–4^. More recently, insufficient or prolonged sleep has been identified as a predictor of adverse health outcomes^5,6^. Generally, lifestyle behaviors have complex relationships and they tend to cluster in specific combinations within populations^7,8^. Moreover, having multiple lifestyle risk factors can have synergistic effects on diseases. Thus, it is important to evaluate the combined effects of lifestyle factors on health outcomes to quantify disease burden and provide valuable public health messages for disease prevention.

A number of epidemiological studies have examined the combined association of major lifestyle factors including obesity, smoking, alcohol, and physical activity in relation to mortality. A systematic review and meta-analysis of 15 cohort studies showed that adherence to at least four healthy lifestyle behaviors was associated with a 66% reduced risk of all-cause mortality, although high heterogeneity (I^2^ = 94%) was observed between study populations^9^. Subsequent studies consistently suggested the importance of healthy lifestyle behaviors for the prevention of diseases^10–18^. However, majority of the studies were conducted in Western populations (e.g., US and Europe). Limited data are available for non-Caucasians, especially Asians^16,19–21^ including Koreans^10,12,18^ whose lifestyle patterns are different from Western populations^22,23^. In addition, these studies had relatively small sample size and restricted study population or did not consider emerging lifestyle factors such as insufficient or prolonged sleep. Recent Korean studies also showed that major lifestyle risk factors were clustered in specific combinations for which the patterns varied by demographic/socioeconomic factors^7,24^.

We therefore used a large nationally representative cohort of Koreans to examine the combined association of 5 major lifestyle risk factors, including unhealthy weight, smoking, alcohol use, physical inactivity and insufficient/prolonged sleep, in relation to all-cause, cardiovascular and cancer mortality. We also examined whether the association between lifestyle risk factors and mortality differs by demographic and socioeconomic factors. Lastly, we further explored different combinations of lifestyle risk factors in relation to mortality.

## Methods

### Study population and database information

The Korea National Health and Nutrition Examination Survey (KNHANES) is a nationally representative, cross-sectional survey which has been carried out since 1998 by the Korea Centers for Disease Control and Prevention (KCDC) to monitor health and nutritional status of Korean citizens^25^. The details of KNHANES are described elsewhere^26^. Briefly, the survey includes three parts: health examination, health interview, and nutrition survey. Upon participants’ consent, the KNHANES 2007–2015 data were linked to death certificates and medical records from January 1, 2007 to December 31, 2016. All participants provided informed consent and the survey was approved by the Ethics Committee of the KCDC (2007-02CON-04-P, 2008-04EXP-01-C, 2009-01COM-03-2C, 2010-02CON-21-C, 2011-02CON-06-C, 2012-01EXP-01-2C, 2013-07CON-03-4C, and 2013-12EXP-03-5C). All methods were performed in accordance with relevant guidelines and regulations.

To allow at least two years of follow-up, we restricted the analysis to the KNHANES 2007–2014 participants. The current analysis included the KNHANES participants who responded to the survey from 2007 to 2014 and consented to mortality follow-up. Among 59,559 participants, we excluded participants who aged <19 years (n = 14,252), those who had a history of cancer (n = 1,349) or cardiovascular disease (n = 1,855), those with missing information on lifestyle risk factors (unhealthy weight, smoking, alcohol use, physical inactivity and insufficient/prolonged sleep) (n = 4,520), and those who died during the first year of follow-up (n = 111). Participants with missing information on lifestyle risk factors had similar characteristics with those included in this study (Supplementary Table 1). As a result, a total of 37,472 individuals (15,827 men, 21,645 women) were included in this study.

### Mortality assessment

Date and causes of death from January 1, 2007 to December 31, 2016 were ascertained by reviewing death certificates and medical records. Using the *International Classification of Disease*, 10^th^ version (ICD-10), we identified all-cause mortality (n = 1,057), deaths from cardiovascular diseases (I00-I99) (n = 213) and cancers (C00-D48) (n = 347).

### Lifestyle risk score

Lifestyle risk score was calculated based on the information from five different lifestyle risk factors (current smoking, high-risk alcohol drinking, unhealthy weight, physical inactivity, insufficient/prolonged sleep)^13^ that have been associated with increased risks of chronic diseases and mortality. Height and weight were measured at physical examination. Body mass index (BMI) was calculated by weight (kg) divided by height squared (m^2^) and those with BMI < 18.5 or ≥25 kg/m^2^ were considered unhealthy weight based on the Asia-Pacific regional guidelines of the World Health Organization^27^. Other lifestyle factors were assessed via health interview. Current smokers were defined as those who have smoked ≥100 cigarettes (five packs) in lifetime and reported as a current regular smoker. To rule out recent initiators, we used ≥100 cigarettes criteria to define current smokers. High-risk alcohol drinking was defined as drinking ≥14 drinks/wk for men and ≥10 drinks/wk for women during the past year^13,28^. Physical activity was assessed using International Physical Activity Questionnaire (IPAQ)^29^ in 2005–2013 and Global Physical Activity Questionnaire (GPAQ) since 2014, which asked the average weekly total time spent on walking, moderate-intensity, and vigorous-intensity physical activity. Participants who engaged in at least 150 min/wk of moderate-intensity activity, at least 75 min/wk of vigorous-intensity activity, or a combination of moderate- and vigorous-intensity activity were considered engaging in sufficient physical activity following the national guideline^30^, and those who did not meet this criteria were considered having insufficient physical activity. Insufficient/prolonged sleep (<7 or ≥9 hr/d) was defined using 7–8 hr/d sleep in the past 24 hours as the reference point^31^. For each of the five selected lifestyle risk factors, participants received a score of 1 if they practiced the unhealthy behavior, otherwise received a score of 0. A total lifestyle risk score ranged from 0 to 5, indicating the sum of these five scores. Higher scores indicate an unhealthier lifestyle. Because information on only few dietary factors was available for the current mortality follow-up study, we did not include dietary factors in the calculation of lifestyle risk score. In secondary analyses only, we further considered excess sodium and total dietary fat intakes (assessed via a single 24-hour recall), separately, as a dietary risk factor in score calculation. Excess sodium intake was defined as ≥2000 mg of sodium intake per day following the World Health Organization recommendation. Excess total dietary fat intake was defined as >25% of total calorie intake from dietary fat according to the Ministry of Food and Drug Safety in Korea. In these secondary analyses, the lifestyle risk score ranged from 0 to 6.

### Statistical analysis

Participants were followed from the survey (baseline) to the date of death or to the end of follow-up on December 31, 2016, whichever occurred first. We performed Cox proportional hazards models, with age (in month) as the time metric and stratifying by the calendar year of survey, to estimate hazard ratios (HRs) and 95% confidence intervals (CIs) for the relationships between the lifestyle risk score (0, 1, 2, 3, 4–5) and all-cause and cause-specific mortality. Because less than 1% of participants had all 5 lifestyle risk factors, we combined lifestyle risk scores 4 and 5. All models were adjusted for potential confounders: sex (men, women), household income (in quartiles, missing), education (<highs school, high school, college or higher, missing), occupation (white collar, blue collar, unemployed/other, missing), residential area (metropolitan area, small cities, rural areas), and marital status (married/live with a partner, unmarried/separated, missing). Potential confounders were selected *a priori* based on the literature on lifestyle and mortality^7,9,24^. Standard errors were adjusted for complex sampling design using sandwich robust variance estimation method. The proportional hazards assumption was tested by time-dependent covariate analysis. Wald test for continuous variable was used to investigate whether there was a linear trend in the association between lifestyle risk score and mortality. The Kaplan-Meier method was used to estimate cumulative mortality according to lifestyle risk score. To examine whether the association is driven by one of the five lifestyle factors that contributed to the lifestyle risk score, we compared models excluding one lifestyle factor at a time while adjusting for the excluded factor. Moreover, we conducted stratified analyses by demographic and socioeconomic characteristics (sex, age, education, income level, occupation, residential area, and marital status) and tested for interaction. In secondary analyses, we repeated the analyses after adding dietary factors (excess sodium or total dietary fat intake) in calculating lifestyle risk score and using different definition for sleep as a risk factor (considering insufficient sleep or prolonged sleep only, without grouping them together). To reduce reverse causation by subclinical diseases, we conducted a sensitivity analysis further excluding deaths occurred during the first 3 years of follow-up (n = 448). To further explore different patterns of lifestyle risk factors, we created all possible mutually exclusive combinations of the five lifestyle factors and examined their relationships with all-cause mortality.

## Results

An average duration of follow-up was 6.01 years. Table 1 shows the baseline characteristics of study participants according to lifestyle risk score. Approximately 19% of the participants had zero lifestyle risk factor and 3% had 4 or 5 lifestyle risk factors. Individuals with higher lifestyle risk score were more likely to be male, younger, and employed. Among the five selected lifestyle risk factors, the most prevalent lifestyle risk factor was insufficient/prolonged sleep (49.3%) followed by unhealthy weight (36.1%), inadequate physical activity (25.3%), smoking (21.3%) and high-risk alcohol drinking (11.5%). The Pearson correlation coefficients among the five lifestyle risk factors ranged from 0.001 to 0.28, with the highest correlation between alcohol drinking and smoking (Supplementary Table 2).

**Table 1:** Baseline characteristics of study participants according to lifestyle risk score in the Korea National Health and Nutrition Examination Surveys 2007–2014.

		Total	Lifestyle risk score									
			0		1		2		3		4–5	
		N	N	%	N	%	N	%	N	%	N	%
Sex	Men	15827	2015	12.7	5040	31.8	5096	32.2	2764	17.5	912	5.8
	Women	21645	4897	22.6	9046	41.8	5855	27.1	1665	7.7	182	0.8
Age group	19–44	16620	3455	20.8	6088	36.6	4400	26.5	2042	12.3	635	3.8
	45–64	13299	2420	18.2	5021	37.8	4005	30.1	1508	11.3	345	2.6
	65+	7553	1037	13.7	2977	39.4	2546	33.7	879	11.6	114	1.5
Education	<High school	12825	1776	13.9	4857	37.9	4282	33.4	1604	12.5	306	2.4
	High school	13185	2615	19.8	4910	37.2	3663	27.8	1580	12.0	417	3.2
	College or higher	11407	2515	22.1	4307	37.8	2986	26.2	1231	10.8	368	3.2
	Missing	55	6	10.9	12	21.8	20	36.4	14	25.5	3	5.5
Income	Q1	6778	921	13.6	2492	36.8	2329	34.4	866	12.8	170	2.5
	Q2-Q3	19749	3640	18.4	7427	37.6	5689	28.8	2378	12.0	615	3.1
	Q4	10505	2288	21.8	3987	38.0	2808	26.7	1129	10.8	293	2.8
	Missing	440	63	14.3	180	40.9	125	28.4	56	12.7	16	3.6
Occupation	White collar	7971	1560	19.6	2918	36.6	2170	27.2	996	12.5	327	4.1
	Blue collar	14707	2339	15.9	5244	35.7	4571	31.1	2011	13.7	542	3.7
	Unemployed	14645	2996	20.5	5881	40.2	4160	28.4	1391	9.5	217	1.5
	Missing	149	17	11.4	43	28.9	50	33.6	31	20.8	8	5.4
Residential area	Metropolitan area	23808	4527	19.0	9052	38.0	6740	28.3	2778	11.7	711	3.0
	Small cities	6368	1236	19.4	2337	36.7	1914	30.1	729	11.5	152	2.4
	Rural areas	7296	1149	15.8	2697	37.0	2297	31.5	922	12.6	231	3.2
Marital status	Married	26950	5125	19.0	10192	37.8	7789	28.9	3083	11.4	761	2.8
	Single, divorced, separated, widowed	10446	1774	17.0	3864	37.0	3143	30.1	1332	12.8	333	3.2
	Missing	76	13	17.1	30	39.5	19	25.0	14	18.4	0	0.0
*Sub-indicators of lifestyle scores (0* = *no, 1* = *yes)*												
Current smoking	0	29493	6912	23.4	12648	42.9	7914	26.8	1911	6.5	108	0.4
	1	7979	0	0.0	1438	18.0	3037	38.1	2518	31.6	986	12.4
High-risk alcohol drinking	0	33167	6912	20.8	13642	41.1	9510	28.7	2823	8.5	280	0.8
	1	4305	0	0.0	444	10.3	1441	33.5	1606	37.3	814	18.9
Unhealthy weight	0	23945	6912	28.9	10573	44.2	5081	21.2	1256	5.3	123	0.5
	1	13527	0	0.0	3513	26.0	5870	43.4	3173	23.5	971	7.2
Inadequate physical activity	0	28000	6912	24.7	11725	41.9	6968	24.9	2026	7.2	369	1.3
	1	9472	0	0.0	2361	24.9	3983	42.1	2403	25.4	725	7.7
Insufficient/prolonged sleep	0	19013	6912	36.4	7756	40.8	3380	17.8	842	4.4	123	0.7
	1	18459	0	0.0	6330	34.3	7571	41.0	3587	19.4	971	5.3
Total		37472	6912	18.5	14086	37.6	10951	29.2	4429	11.8	1094	2.9

**Figure 1:**
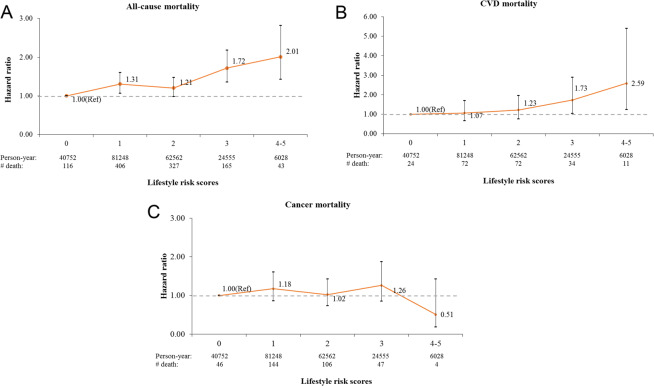
Associations of lifestyle risk score with (**A**) all-cause, (**B**) cardiovascular disease, and (**C**) cancer mortality in the Korea National Health and Nutrition Examination Surveys 2007–2014. All models were adjusted for sex, age, education, income, occupation, regional area, and marital status.

Higher lifestyle risk score was significantly positively associated with all-cause (4–5 vs. 0: HR = 2.01, 95% CI = 1.43–2.82, p-trend < 0.001; Fig. 1A) and CVD mortality (4–5 vs. 0: HR = 2.59, 95% CI = 1.24–5.40, p-trend = 0.004; Fig. 1B) but not associated with cancer mortality (4–5 vs. 0: HR = 0.51, 95% CI = 0.18–1.43, p-trend = 0.82; Fig. 1C). The Kaplan-Meier curves for cumulative all-cause and cause-specific mortality showed consistent results (Supplementary Fig. 1). When we further included sodium intake or total dietary fat intake in the score calculation, few participants practiced 5 or more lifestyle risk factors but we consistently observed a positive association with all-cause mortality (Supplementary Table 3). Moreover, we found similar positive associations when using only ‘insufficient sleep’ (or ‘prolonged sleep’) instead of ‘insufficient or prolonged sleep’ (Supplementary 4).

Table 2 presents the associations between lifestyle risk score and all-cause mortality after excluding one lifestyle factor at a time. We found a statistically significant, linear positive trend with scores excluding high-risk alcohol drinking (p-trend < 0.001), unhealthy weight (p-trend < 0.001), physical activity (p-trend=0.004), or sleep duration (p-trend < 0.001). The magnitude of the positive associations became weaker after excluding current smoking, high-risk alcohol drinking, inadequate physical activity, and sleep duration.

**Table 2:** Hazard ratios (HRs) and 95% confidence intervals (CIs) for the associations between lifestyle risk score and all-cause mortality in the Korea National Health and Nutrition Examination Surveys 2007–2014.

	Lifestyle risk score				
	0	1	2	3–4	p-trend
**Excluding current smoking**					
Death	177	451	311	118	
Person-year	49326	90684	59082	16054	
HR (95% CI)	1.00 (Ref)	1.11 (0.93–1.33)	1.04 (0.87–1.25)	1.38 (1.09–1.75)	0.08
**Excluding high-risk alcohol drinking**					
Death	127	423	343	164	
Person-year	43486	87185	63229	21246	
HR (95% CI)	1.00 (Ref)	1.25 (1.04–1.51)	1.23 (1.01–1.50)	1.66 (1.33–2.08)	<0.001
**Excluding unhealthy weight**					
Death	187	471	294	105	
Person-year	61440	94797	45891	13018	
HR (95% CI)	1.00 (Ref)	1.21 (1.03–1.42)	1.39 (1.16–1.67)	2.14 (1.67–2.72)	<0.001
**Excluding inadequate physical activity**					
Death	177	490	290	100	
Person-year	53408	89431	54171	18136	
HR (95% CI)	1.00 (Ref)	1.11 (0.94–1.32)	1.13 (0.94–1.35)	1.59 (1.24–2.04)	0.004
**Excluding insufficient/prolonged sleep**					
Death	318	443	238	58	
Person-year	77348	88022	38978	10798	
HR (95% CI)	1.00 (Ref)	1.11 (0.96–1.29)	1.42 (1.19–1.69)	1.61 (1.22–2.13)	<0.001

Adjusted for sex, age, education, income, occupation, regional area, marital status, and the excluded factor.

Table 3 shows the results from stratified analyses by sex, age, education level, income level, occupation, residential area, and marital status. We observed stronger associations in adults <65 years (vs. adults ≥65 years), lower (vs. higher) education levels, unemployed (vs. employed), and single/divorced/separated (vs. married). However, the interactions were only statistically significant for education level, occupation, and marital status (p-interaction < 0.001 for all).

**Table 3:** Hazard ratios (HRs) and 95% confidence intervals (CIs) for the associations between lifestyle risk score and all-cause mortality, stratified by demographic and socioeconomic factors.

		Lifestyle risk score																									
		0					1					2					3					4–5					p-inter-action
		PY	N	HR	95% CI		PY	N	HR	95% CI		PY	N	HR	95% CI		PY	N	HR	95% CI		PY	N	HR	95% CI		
**Sex**																											
Men		11629	64	1.00			28787	233	1.48	1.13	1.93	29735	189	1.34	1.02	1.76	15692	104	1.88	1.40	2.53	5038	33	2.03	1.36	3.04	0.22
Women		29123	52	1.00			52462	173	1.10	0.82	1.47	32827	138	1.03	0.75	1.41	8863	61	1.47	1.00	2.16	990	10	3.30	1.66	6.57	
**Age group**																											
19–44		20843	10	1.00			35761	25	1.38	0.66	2.89	25946	16	0.99	0.43	2.25	11829	12	1.41	0.56	3.58	3603	10	3.31	1.27	8.67	0.14
45–64		14050	17	1.00			29073	65	1.63	0.96	2.76	22814	66	1.81	1.06	3.10	8259	43	2.75	1.56	4.82	1826	11	3.25	1.55	6.79	
65+		5859	89	1.00			16414	316	1.23	0.99	1.54	13802	245	1.11	0.88	1.40	4467	110	1.53	1.15	2.05	600	22	1.50	0.96	2.35	
**Education**																											
<High school		10719	76	1.00			28359	304	1.33	1.04	1.70	24305	256	1.25	0.97	1.61	8691	128	1.78	1.33	2.38	1687	34	2.05	1.37	3.06	<.001
High school		15428	25	1.00			28694	72	1.54	0.95	2.50	21106	47	1.24	0.76	2.01	8874	26	2.01	1.14	3.52	2325	7	2.16	0.94	4.99	
College or higher		14573	15	1.00			24127	26	0.92	0.47	1.79	17042	24	1.23	0.62	2.41	6906	9	1.18	0.46	2.99	1993	2	0.93	0.17	5.20	
**Income (quartiles)**																											
Q1		5526	56	1.00			14421	180	1.09	0.80	1.47	13106	166	1.10	0.82	1.49	4593	87	1.76	1.24	2.50	963	29	2.30	1.50	3.53	0.09
Q2-Q3		21427	41	1.00			42643	173	1.58	1.14	2.19	32365	109	1.28	0.91	1.81	13211	60	1.69	1.13	2.53	3362	6	0.85	0.34	2.09	
Q4		13377	18	1.00			22952	43	1.08	0.62	1.89	16251	44	1.24	0.72	2.13	6374	14	1.22	0.56	2.63	1606	6	2.67	0.93	7.67	
**Occupation**																											
White collar		8952	8	1.00			16454	12	0.55	0.21	1.46	12399	16	0.88	0.35	2.22	5643	7	1.09	0.32	3.69	1777	2	1.12	0.22	5.86	<.001
Blue collar		14058	45	1.00			30910	144	1.30	0.94	1.80	26557	106	1.14	0.82	1.56	11342	54	1.58	1.05	2.36	3011	13	1.76	0.93	3.31	
Unemployed		17624	63	1.00			33607	246	1.38	1.06	1.81	23277	205	1.28	0.97	1.69	7369	102	1.86	1.36	2.56	1180	28	2.24	1.38	3.62	
**Residential area**																											
Metropolitans		26125	47	1.00			51517	203	1.65	1.21	2.24	38125	163	1.54	1.12	2.11	15421	85	2.17	1.51	3.12	3901	21	2.63	1.58	4.37	0.05
Small cities		7423	21	1.00			13503	67	1.37	0.82	2.28	11069	43	0.88	0.52	1.49	4036	33	2.23	1.38	3.61	866	5	1.75	0.53	5.74	
Rural areas		7204	48	1.00			16229	136	0.99	0.72	1.36	13369	121	1.04	0.74	1.45	5098	47	1.16	0.76	1.78	1261	17	1.89	1.17	3.06	
**Marital status**																											
Married		30331	86	1.00			58789	276	1.36	1.07	1.74	44670	203	1.22	0.96	1.55	17155	105	1.83	1.37	2.45	4151	24	1.87	1.22	2.87	<.001
Single, divorced, separated, widowed		10323	30	1.00			22281	129	1.21	0.82	1.79	17764	123	1.19	0.79	1.79	7300	60	1.52	0.99	2.34	1878	19	2.49	1.33	4.65	

Adjusted for sex, age, education, income, occupation, regional area, and marital status, except for the stratum itself.

We also examined all combinations of five lifestyle risk factors and their associations with mortality (Supplementary Table 5). Among participants with 2 or more risk factors, the most common combinations were unhealthy weight + insufficient/prolonged sleep (9.5%), followed by inadequate physical activity + insufficient/prolonged sleep (6.1%). Among single lifestyle risk factors, the strongest positive association with all-cause mortality was shown for smoking (HR = 1.89), followed by inadequate physical activity (HR = 1.56). Among multiple lifestyle risk factor combinations, several combinations tended to show stronger associations with all-cause mortality: smoking + high-risk alcohol drinking + insufficient/prolonged sleep (HR = 2.49), high-risk alcohol drinking + inadequate physical activity + insufficient/prolonged sleep (HR = 2.49), and smoking + high-risk alcohol drinking + inadequate physical activity + insufficient/prolonged sleep (HR = 3.28). In a sensitivity analysis excluding deaths occurred during the first 3 years of follow-up, the association between lifestyle risk score and all-cause and cause-specific mortality did not change materially (Supplementary Table 6).

## Discussion

Among 37,472 Korean men and women, having more unhealthy lifestyle risk factors was associated with considerably higher risk of mortality. Compared to Korean adults with no unhealthy lifestyle habits, those with 4 to 5 unhealthy lifestyle habits had a 2-fold higher risk of all-cause mortality and a 2.6-fold higher risk of cardiovascular mortality. In contrast, we found no association between lifestyle risk factors and cancer mortality among Korean adults. To our knowledge, this is the first study to investigate the combined impact of major lifestyle factors on all-cause and cause-specific mortality using a large nationally representative sample of Korean adults.

A large body of existing evidence shows that being obese, physically inactive, smoking and heavy alcohol use respectively have harmful effects on diverse diseases and overall health^1–4^. However, relatively fewer studies have examined the combined effects of these major lifestyle factors on health outcomes^9–18^. Moreover, most of the existing studies have been conducted among Western populations (i.e., Caucasians) and found a strong inverse association between a combined healthy lifestyle habits and overall mortality. On the other hand, we identified a limited number of studies from Asian populations^16,19–21^ including three Korean studies^10,12,18^. A large prospective cohort followed Korean adults who participated in a medical examination at the Severance Health Promotion Center and found that having a combination of four unhealthy lifestyle factors (obesity, smoking, alcohol, physical inactivity) was associated with approximately 2-fold increased risk of total mortality^10^. This study did not specifically examine cardiovascular mortality, but they found similar magnitude of the associations for cancer and non-cancer mortality. Another study that included 9,945 Koreans with an average age of 60 years from the Korean Longitudinal Study of Aging reported similar results: compared to participants with no lifestyle risk factor, those with three or more lifestyle risk factors had 3.5- and 5.4-fold higher risk of total and cardiovascular mortality, respectively^18^. The Seoul Male Cohort Study used a modified lifestyle score and examined the association of 7 cardiovascular health metrics, including BMI, smoking, physical activity and diet, with mortality among middle-aged Korean men^12^. Although the factors included in the metric was not directly comparable to other studies, this study also suggested that healthy lifestyle habits were associated with markedly lower risk of total and cardiovascular mortality.

Beyond the traditional lifestyle factors including obesity, smoking, alcohol, and physical inactivity, our study additionally considered an emerging risk factor (‘insufficient or prolonged sleep’) in the lifestyle risk score. Recent studies have shown convincing evidence that insufficient or prolonged sleep (e.g., 6≤ or ≥10 hours) is associated with a number of chronic diseases including diabetes, cardiovascular and cancers^5,6^. Two cohort studies from Japan^20^ and Australia^13^ examined the combined impact of lifestyle risk behaviors including short or long sleep duration and found a positive association with mortality. Interestingly, in the Australian study, lifestyle patterns that included insufficient/prolonged sleep (e.g., physical inactivity + prolonged sitting time + long sleep/smoking + high alcohol + short sleep) showed relatively stronger positive associations than the other common risk combinations^13^.

In our secondary analyses, we consistently found a positive association between combined lifestyle risk score and mortality after excluding each lifestyle risk factor from the score. However, we observed greater attenuation of the association when smoking was excluded from the score, indicating that smoking is more deleterious than other lifestyle risk behaviors. We also found similar patterns when we explored different combinations of five lifestyle risk factors in relation to mortality. Among those with the same number of lifestyle risk behaviors, participants who had smoking as one of their lifestyle risk behavior tended to have higher risk of mortality. Our findings suggest that smoking cessation could be more effective strategy than other healthy lifestyle behaviors. Nevertheless, we clearly observed increased mortality risk with higher number of lifestyle risk behaviors and thus it is important to emphasize adopting overall healthy lifestyle behaviors rather than focusing on single factors to maximize the prevention of disease and premature death.

Although we considered five important lifestyle factors, our lifestyle risk score has some limitations. We did not have sufficient dietary information to evaluate overall diet quality, which is also an important lifestyle factor. Several studies have demonstrated that healthy dietary patterns were associated with lower risk of coronary heart disease and mortality in Korean adults^32–34^. Our findings (i.e., lifestyle risk score) do not fully capture the additional benefits of maintaining healthy diets. With the limited dietary information (a single 24-hour recall only), we conducted a secondary analysis further including ‘high sodium or total dietary fat intake’ as a proxy of poor diets. In this analysis, we observed a stronger but non-significant positive association with total mortality, which is likely due to a small number of participants with all six lifestyle risk factors. Given the growing number of studies showing the important role of diet^35^ and other modifiable factors (e.g., prolonged sitting)^13^ on health, more studies are needed to examine the association of comprehensive lifestyle risk score including diet quality and emerging risk factors in relation to mortality in Korean adults.

When we conducted stratified analysis, the positive association between combined lifestyle risk factors and mortality tended to be stronger in adults aged <65 years and participants with lower education and unemployed status. Intriguingly, a previous study indicated that the aforementioned groups are more susceptible to having multiple lifestyle risk factors^7^. These findings suggest that adherence to healthy lifestyle habits may also have greater impact on disease prevention for younger adults or those with lower socioeconomic status. There were very limited studies that thoroughly explored the associations by demographic/socioeconomic factors in Korean or other populations. This is most likely due to restricted study population and limited sample size of individual studies to perform adequate subgroup analyses. From public health perspective, it is critical to identify individuals who are more vulnerable to adverse health-related conditions that are attributable to unhealthy lifestyle behaviors for more effective and efficient lifestyle intervention targeting.

There are several strengths of the current study. First, a prospective design of the study minimizes the concern of differential misclassification. Moreover, we excluded deaths occurred during the first year of follow-up, which further reduced bias due to reverse causation. Second, this is one of few data from Asian populations and the first Korean study to examine the combined association of lifestyle risk factors and mortality using a large nationally representative sample of Koreans. Findings from this study has high generalizability. Third, we included five lifestyle risk factors including four traditional and one emerging risk factors, and they were assessed using validated methods by trained personnel. This study has limitations as well. We only used baseline lifestyle factors, and hence we were not able to capture the changes in lifestyle factors over the follow-up period. Moreover, we had a relatively short follow-up period (mean of 6 years) and limited number of deaths. Some of our analyses did not have enough power to detect significant results. Especially, our null finding for cancer mortality, which is contrary to previous studies that showed an inverse association between healthy lifestyles and cancer mortality^36^, can be due to limited follow-up time and number of cancer deaths, or by chance and thus should be interpreted with caution. More prospective studies with longer follow-up period are needed to reexamine cause-specific mortality in this population. Lastly, although we carefully adjusted for potential confounders, we cannot completely rule out the possibility of residual confounding by unmeasured factors.

In conclusion, we found that unhealthy lifestyle behaviors, including smoking, heavy alcohol use, obesity, physical inactivity and insufficient/prolonged sleep, in combination were strongly associated with increased risks of total and cardiovascular mortality in Korean men and women. Interventions and strategies promoting multiple healthy lifestyle behaviors may have substantial implications to reduce chronic diseases and premature death among Korean adults.

## Supplementary information

Supplementary information.

## Data Availability

The datasets generated during and/or analyzed during the current study are available from the corresponding author on a reasonable request.
