# Supplementary material for: Lifestyle risk score and mortality in Korean adults: a population-based cohort study
Source: Sci Rep. 2020 Jun 24;10:10260. doi: 10.1038/s41598-020-66742-y (PMC7314763; doi:10.1038/s41598-020-66742-y)

**Supplementary Materials**

**Lifestyle risk score and mortality in Korean adults: a population-based cohort study**

Dong Hoon Lee^1*^, Jin Young Nam^2*^, Sohyeon Kwon^2^, NaNa Keum^1,3^, Jong-Tae Lee^2,4^, Min-Jeong Shin^2,5^, Hannah Oh^2,4^

^1^ Department of Nutrition, Harvard T.H. Chan School of Public Health, Boston, MA, USA

^2^ Department of Public Health Sciences, BK21PLUSProgram in Embodiment: Health-Society Interaction, Graduate School, Korea University, Seoul, Republic of Korea

^3^ Department of Food Science and Biotechnology, Dongguk University, Goyang, South Korea

^4^ Division of Health Policy and Management, College of Health Sciences, Korea University, Seoul, Republic of Korea

^5^ Division of Biosystems and Biomedical Sciences, College of Health Sciences, Korea University, Seoul, Republic of Korea

| **Supplementary Table 1. General participant characteristics according to lifestyle score with missing vs non-missing value from 2007 to 2014** | | | | | |
| --- | --- | --- | --- | --- | --- |
|  |  | **Missing** | | **Non-missing** | |
|  |  | N | % | N | % |
| Lifestyle score | 0 | 437 | 9.73 | 6912 | 18.5 |
|  | 1 | 1066 | 23.75 | 14086 | 35.6 |
|  | 2 | 1894 | 42.19 | 10951 | 29.2 |
|  | 3 | 991 | 22.08 | 4429 | 11.8 |
|  | 4-5 | 101 | 2.25 | 1094 | 2.9 |
| Sex | Men | 1956 | 43.6 | 15827 | 42.2 |
|  | Women | 2533 | 56.4 | 21645 | 57.8 |
| Age group | 19-44 | 1753 | 39.1 | 16620 | 44.4 |
|  | 45-64 | 1495 | 33.3 | 13299 | 35.5 |
|  | 65+ | 1241 | 27.7 | 7553 | 20.2 |
| Education | < High school | 1181 | 26.3 | 12825 | 34.2 |
|  | High school | 932 | 20.8 | 13185 | 35.2 |
|  | College or higher | 689 | 15.4 | 11407 | 30.4 |
|  | Missing | 1687 | 37.6 | 55 | 0.2 |
| Income | Q1 | 1018 | 22.7 | 6778 | 18.1 |
|  | Q2-Q3 | 2179 | 48.5 | 19749 | 52.7 |
|  | Q4 | 1037 | 23.1 | 10505 | 28.0 |
|  | Missing | 255 | 5.7 | 440 | 1.2 |
| Occupation | White collar | 498 | 11.1 | 7971 | 21.3 |
|  | Blue collar | 988 | 22.0 | 14707 | 39.3 |
|  | Unemployed | 1268 | 28.3 | 14645 | 39.1 |
|  | Missing | 1735 | 38.7 | 149 | 0.4 |
| Residential area | Metropolitan area | 2669 | 59.5 | 23808 | 63.5 |
|  | Small cities | 692 | 15.4 | 6368 | 17.0 |
|  | Rural areas | 1128 | 25.1 | 7296 | 19.5 |
| Marital status | Married | 3105 | 69.2 | 26950 | 71.9 |
|  | Single, divorced, separated, widowed | 1237 | 27.6 | 10446 | 27.9 |
|  | Missing | 147 | 3.3 | 76 | 0.2 |

| **Supplementary Table 2. The Pearson correlation coefficient among lifestyle risk behavior factors**. | | | | | | |
| --- | --- | --- | --- | --- | --- | --- |
|  |  | Smoking | Alcohol intake | Unhealthy Weight | Inadequate physical activity | Insufficient/prolonged sleep |
| Total | Smoking | 1 |  |  |  |  |
|  | Alcohol consumption | 0.276^***^ | 1 |  |  |  |
|  | Unhealthy Weight | 0.023^***^ | 0.05^***^ | 1 |  |  |
|  | Inadequate physical activity | -0.018^***^ | -0.03^***^ | 0.002 | 1 |  |
|  | Insufficient/ prolonged sleep | -0.003 | 0.004 | 0.027^***^ | 0.015^**^ | 1 |
| Men | Smoking | 1 |  |  |  |  |
|  | Alcohol consumption | 0.175^***^ | 1 |  |  |  |
|  | Unhealthy Weight | -0.001 | 0.067^***^ | 1 |  |  |
|  | Inadequate physical activity | 0.042^***^ | -0.013 | -0.003 | 1 |  |
|  | Insufficient/ prolonged sleep | -0.012 | 0.008 | 0.016^*^ | 0.009 | 1 |
| Women | Smoking | 1 |  |  |  |  |
|  | Alcohol consumption | 0.219^***^ | 1 |  |  |  |
|  | Unhealthy Weight | -0.001 | -0.002 | 1 |  |  |
|  | Inadequate physical activity | 0.006 | -0.002 | 0.013 | 1 |  |
|  | Insufficient/ prolonged sleep | 0.028^***^ | 0.010 | 0.036^***^ | 0.018^**^ | 1 |
| ^*^P<0.05, ^**^P<0.01, ^***^P<0.001 | | | | | | |

| **Supplementary Table 3. Hazard ratios (HRs) and 95% confidence intervals (CIs) for the associations between lifestyle risk score and all-cause mortality, after adding sodium and total dietary fat intake into lifestyle risk score calculation** | | | | | | | | | | | |
| --- | --- | --- | --- | --- | --- | --- | --- | --- | --- | --- | --- |
|  |  | **Score with sodium intake ^a^** | | | | | **Score with total dietary fat intake ^b^** | | | | |
|  |  | **PY** | **N** | **HR** | **95% CI** | | **PY** | **N** | **HR** | **95% CI** | |
| Lifestyle risk score | 0 | 5115 | 20 | 1.00 |  |  | 30578 | 97 | 1.00 |  |  |
|  | 1 | 42757 | 185 | 1.16 | 0.70 | 1.92 | 67293 | 367 | 1.38 | 1.10 | 1.72 |
|  | 2 | 69968 | 345 | 1.17 | 0.72 | 1.89 | 58100 | 302 | 1.24 | 0.99 | 1.56 |
|  | 3 | 49341 | 279 | 1.23 | 0.75 | 2.02 | 25205 | 156 | 1.70 | 1.32 | 2.21 |
|  | 4 | 17964 | 113 | 1.55 | 0.93 | 2.58 | 6902 | 41 | 1.88 | 1.30 | 2.72 |
|  | 5-6 | 4139 | 28 | 2.01 | 1.10 | 3.66 | 1207 | 7 | 3.32 | 1.64 | 6.70 |
| Adjusted for sex, age, educational level, income, occupation, regional area, and marital status.  ^a^ Lifestyle risk score calculated based on current smoking, high-risk alcohol drinking, obesity, physical activity, sleep duration, and sodium intake  ^b^ Lifestyle risk score calculated based on current smoking, high-risk alcohol drinking, obesity, physical activity, sleep duration, and total dietary fat intake | | | | | | | | | | | |

| **Supplementary Table 4. Hazard ratios (HRs) and 95% confidence intervals (CIs) for the associations between lifestyle risk score and all-cause mortality, using different definition of sleep as a risk factor** | | | | | | | | | | |
| --- | --- | --- | --- | --- | --- | --- | --- | --- | --- | --- |
|  |  | **Sleeping for <7 or 9≥h** | | | **Sleeping for <7h** | | | **Sleeping for 9≥h** | | |
|  |  | **HR** | **95% CI** | | **HR** | **95% CI** | | **HR** | **95% CI** | |
| Lifestyle scores | 0 | 1.00 |  |  | 1.00 |  |  | 1.00 |  |  |
|  | 1 | 1.31 | 1.07 | 1.60 | 1.19 | 1.00 | 1.42 | 1.15 | 0.98 | 1.34 |
|  | 2 | 1.21 | 0.98 | 1.48 | 1.08 | 0.89 | 1.31 | 1.43 | 1.20 | 1.70 |
|  | 3 | 1.72 | 1.36 | 2.18 | 1.60 | 1.27 | 2.02 | 1.51 | 1.16 | 1.97 |
|  | 4-5 | 2.01 | 1.43 | 2.82 | 1.50 | 1.01 | 2.24 | 3.20 | 2.04 | 5.02 |

| **Supplementary Table 5. Combination of lifestyle risk behavior factors on mortality from 2007 to 2014** | | | | | | | | | | | | |
| --- | --- | --- | --- | --- | --- | --- | --- | --- | --- | --- | --- | --- |
| **No.** | **Lifestyle risk behavior** | | | | | **Total** | | **All-cause mortality** | | | | |
|  | **SM** | **A** | **W** | **P** | **SP** | **Cohort N** | **%** | **Person-year** | **death N** | **HR*** | **95% CI** | |
| 5 | 1 | 1 | 1 | 1 | 1 | 91 | 0.2 | 457.1 | 4 | 4.74 | 1.66 | 13.52 |
| 4 | 1 | 1 | 1 | 1 | 0 | 123 | 0.3 | 653.7 | 1 | 1.59 | 0.22 | 11.51 |
|  | 1 | 1 | 1 | 0 | 1 | 369 | 1.0 | 2230.0 | 7 | 1.55 | 0.69 | 3.45 |
|  | 1 | 1 | 0 | 1 | 1 | 123 | 0.3 | 680.4 | 8 | 3.28 | 1.20 | 8.94 |
|  | 1 | 0 | 1 | 1 | 1 | 280 | 0.8 | 1459.7 | 21 | 1.99 | 1.29 | 3.08 |
|  | 0 | 1 | 1 | 1 | 1 | 108 | 0.3 | 547.5 | 2 | 1.44 | 0.39 | 5.31 |
| 3 | 1 | 1 | 1 | 0 | 0 | 350 | 0.9 | 2171.4 | 6 | 1.73 | 0.75 | 3.99 |
|  | 1 | 1 | 0 | 1 | 0 | 150 | 0.4 | 804.6 | 4 | 2.39 | 0.92 | 6.22 |
|  | 1 | 1 | 0 | 0 | 1 | 535 | 1.4 | 3199.1 | 20 | 2.49 | 1.45 | 4.26 |
|  | 1 | 0 | 1 | 1 | 0 | 250 | 0.7 | 1346.4 | 4 | 0.91 | 0.36 | 2.29 |
|  | 1 | 0 | 1 | 0 | 1 | 781 | 2.1 | 4648.2 | 24 | 1.67 | 1.07 | 2.62 |
|  | 1 | 0 | 0 | 1 | 1 | 452 | 1.2 | 2359.7 | 32 | 2.22 | 1.45 | 3.40 |
|  | 0 | 1 | 1 | 1 | 0 | 92 | 0.3 | 446.8 | 1 | 1.53 | 0.27 | 8.70 |
|  | 0 | 1 | 1 | 0 | 1 | 360 | 1.0 | 2089.1 | 7 | 1.32 | 0.61 | 2.85 |
|  | 0 | 1 | 0 | 1 | 1 | 119 | 0.3 | 626.2 | 6 | 2.49 | 1.07 | 5.77 |
|  | 0 | 0 | 1 | 1 | 1 | 1340 | 3.6 | 6863.4 | 61 | 1.53 | 1.12 | 2.11 |
| 2 | 1 | 1 | 0 | 0 | 0 | 527 | 1.4 | 3220.6 | 14 | 2.15 | 1.23 | 3.77 |
|  | 1 | 0 | 1 | 0 | 0 | 803 | 2.1 | 4846.1 | 22 | 1.76 | 1.13 | 2.74 |
|  | 1 | 0 | 0 | 1 | 0 | 430 | 1.2 | 2260.6 | 18 | 1.74 | 1.07 | 2.84 |
|  | 1 | 0 | 0 | 0 | 1 | 1277 | 3.4 | 7682.3 | 52 | 1.48 | 1.06 | 2.05 |
|  | 0 | 1 | 1 | 0 | 0 | 346 | 0.9 | 2134.8 | 3 | 0.53 | 0.17 | 1.71 |
|  | 0 | 1 | 0 | 1 | 0 | 126 | 0.3 | 653.0 | 1 | 0.42 | 0.05 | 3.53 |
|  | 0 | 1 | 0 | 0 | 1 | 442 | 1.2 | 2662.2 | 10 | 1.15 | 0.59 | 2.23 |
|  | 0 | 0 | 1 | 1 | 0 | 1148 | 3.1 | 6077.4 | 30 | 1.19 | 0.78 | 1.80 |
|  | 0 | 0 | 1 | 0 | 1 | 3573 | 9.5 | 21178.0 | 81 | 0.89 | 0.68 | 1.18 |
|  | 0 | 0 | 0 | 1 | 1 | 2279 | 6.1 | 11847.5 | 96 | 1.30 | 0.99 | 1.70 |
| 1 | 1 | 0 | 0 | 0 | 0 | 1438 | 3.8 | 8574.0 | 61 | 1.89 | 1.40 | 2.56 |
|  | 0 | 1 | 0 | 0 | 0 | 444 | 1.2 | 2734.1 | 11 | 1.48 | 0.79 | 2.80 |
|  | 0 | 0 | 1 | 0 | 0 | 3513 | 9.4 | 20687.9 | 71 | 1.08 | 0.81 | 1.45 |
|  | 0 | 0 | 0 | 1 | 0 | 2361 | 6.3 | 12656.2 | 61 | 1.56 | 1.14 | 2.13 |
|  | 0 | 0 | 0 | 0 | 1 | 6330 | 16.9 | 36596.3 | 202 | 1.21 | 0.97 | 1.52 |
| 0 | 0 | 0 | 0 | 0 | 0 | 6912 | 18.5 | 40752.2 | 116 | 1.00 |  |  |
| SM, smoking; A, high-risk alcohol drinking; W, unhealthy weigh; P, inadequate physical activity; SP, insufficient/prolonged sleep. | | | | | | | | | | | | |

**Supplementary Table 6. Hazard ratios (HRs) and 95% confidence intervals (CIs) for associations between the all-cause, cancer, and cardiovascular disease mortality and lifestyle risk behaviors excluding deaths within first 3 years.**

|  |  | **All-cause mortality** | | | | | **Cardiovascular disease mortality** | | | | | **Cancer mortality** | | | | |
| --- | --- | --- | --- | --- | --- | --- | --- | --- | --- | --- | --- | --- | --- | --- | --- | --- |
|  |  | **PY** | **N** | **HR** | **95% CI** | | **PY** | **N** | **HR** | **95% CI** | | **PY** | **N** | **HR** | **95% CI** | |
| Lifestyle scores | 0 | 40709 | 94 | 1.00 |  |  | 40709 | 19 | 1.00 |  |  | 40709 | 40 | 1.00 |  |  |
|  | 1 | 80968 | 272 | 1.10 | 0.88 | 1.37 | 80968 | 49 | 0.94 | 0.55 | 1.59 | 80968 | 99 | 0.95 | 0.67 | 1.36 |
|  | 2 | 62334 | 221 | 1.02 | 0.81 | 1.29 | 62334 | 45 | 1.00 | 0.58 | 1.72 | 62334 | 74 | 0.84 | 0.57 | 1.23 |
|  | 3 | 24428 | 104 | 1.43 | 1.08 | 1.90 | 24428 | 25 | 1.74 | 0.97 | 3.11 | 24428 | 29 | 0.94 | 0.58 | 1.51 |
|  | 4-5 | 6001 | 29 | 1.80 | 1.20 | 2.69 | 6001 | 7 | 2.32 | 0.95 | 5.66 | 6001 | 3 | 0.48 | 0.15 | 1.56 |
| P for trend ^a^ |  | 0.01 | | | | | 0.03 | | | | | 0.27 | | | | |
| Adjusted for sex, age, educational level, income, occupation, regional area, and marital status.  ^a^ P for interaction adjusting covariates | | | | | | | | | | | | | | | | |

**Supplementary Figure 1. Cumulative all-cause, cardiovascular disease (CVD), and cancer mortality according to combined lifestyle risk score**

1. All-cause mortality


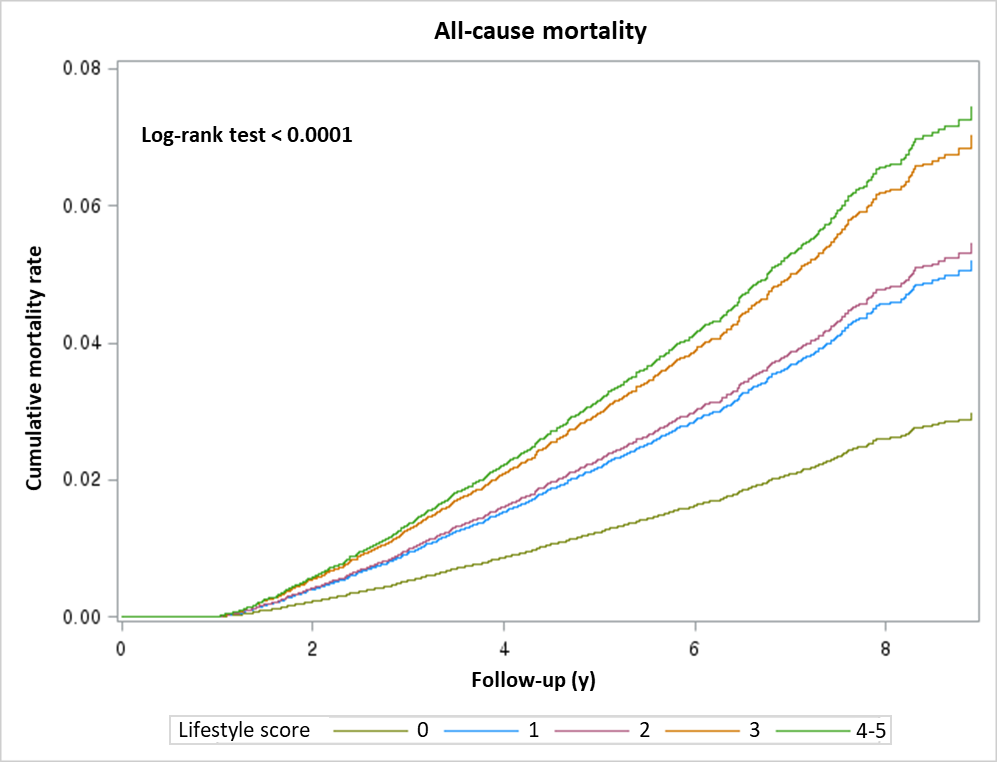


1. CVD mortality


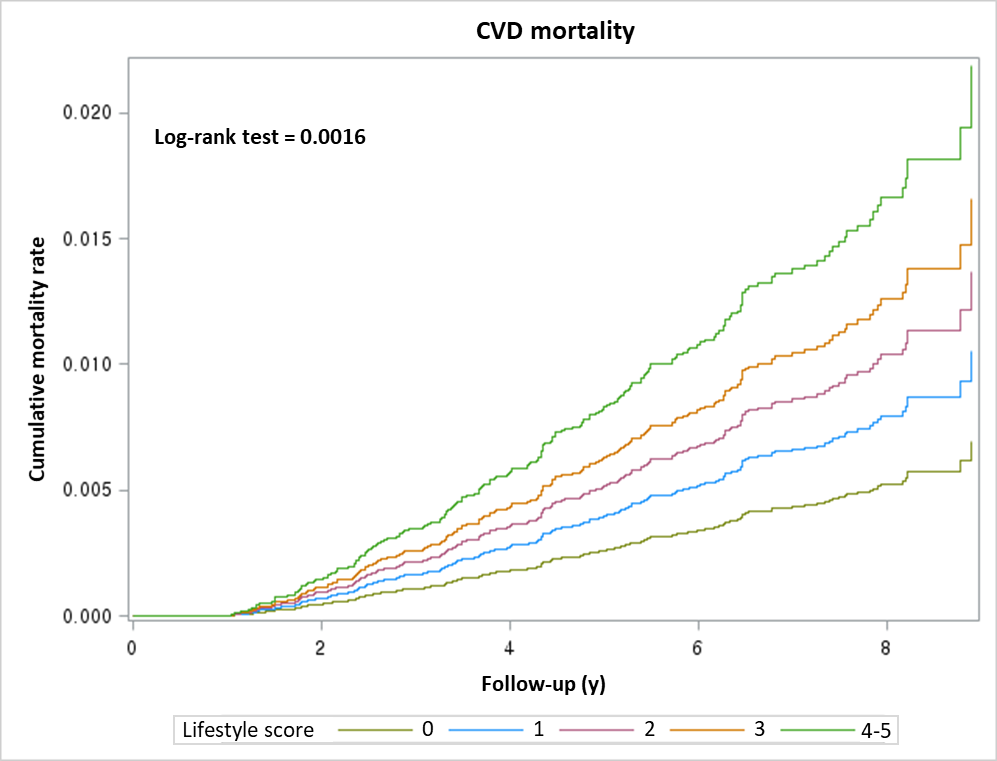


1. Cancer mortality


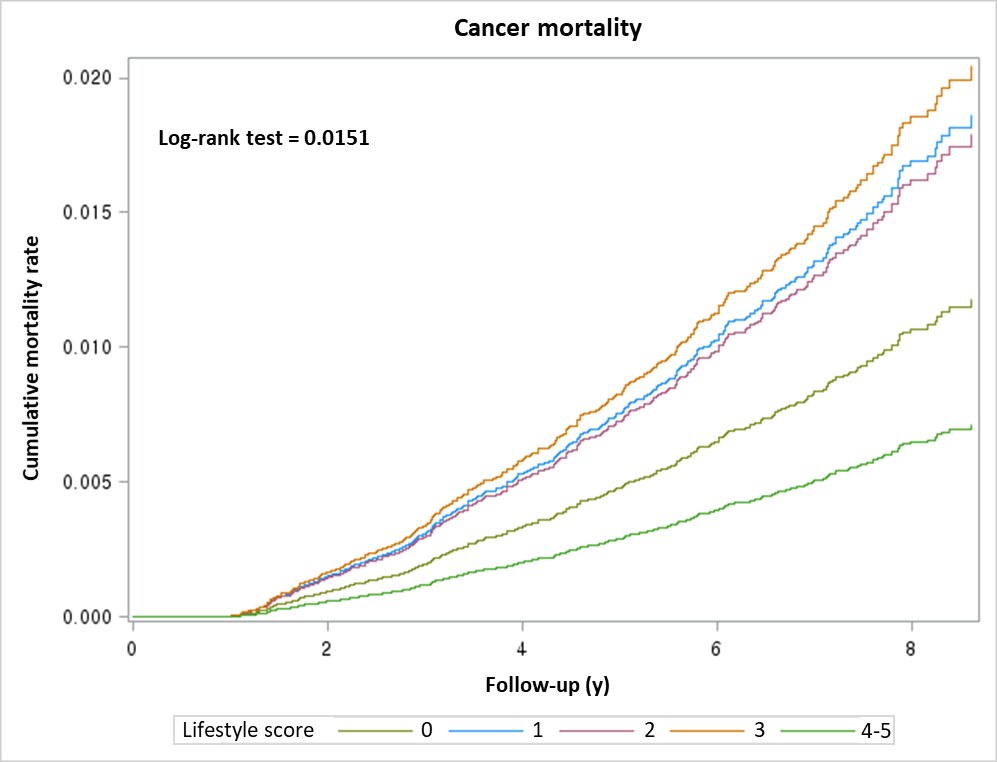

Supplement: Supplementary file 1 — Supplementary information. [file 41598_2020_66742_MOESM1_ESM.docx]
